# Complete genome sequence of a nucleopolyhedrovirus isolated from *Bombyx mori* in Hakozaki, Japan, obtained using Oxford Nanopore Technologies sequencing

**DOI:** 10.1128/mra.01206-23

**Published:** 2024-02-20

**Authors:** Tsuneyuki Tatsuke

**Affiliations:** 1 Division of Silk-Producing Insect Biotechnology, Institute of Agrobiological Sciences, National Agriculture and Food Research Organization, Tsukuba, Ibaraki, Japan; Katholieke Universiteit Leuven, Leuven, Belgium

**Keywords:** baculovirus, insect viruses

## Abstract

I report the complete genome sequence of an isolate of nucleopolyhedrovirus from *Bombyx mori*, the domesticated silkworm, maintained for the long term at Kyushu University in Hakozaki, Fukuoka Prefecture, Japan. The genome is 127,783 bp long, with a G+C content of 40.3%, and contains 142 open reading frames.

## ANNOUNCEMENT

*Bombyx mori* nucleopolyhedrovirus (BmNPV) (family *Baculoviridae*, genus *Alphabaculovirus*) has a 130 kb circular double-stranded DNA genome encoding approximately 140 proteins and is a significant pathogen that specifically infects the domesticated silkworm and the wild mulberry silkworm, causing substantial losses in sericulture (1). On the other hand, it is an industrially important virus used as the baculovirus expression system to produce recombinant proteins from foreign genes using silkworm larvae and pupae (2).

Here, I report the complete genome sequence of a BmNPV strain isolated on 22 May 2015 from the *Bombyx mori* strain k45 fourth-instar larvae maintained at Kyushu University in Hakozaki, Fukuoka Prefecture, Japan, prior to its relocation to Ito in 2018. Numerous mutant strains of silkworms were collected and preserved there from 1924. To understand the relationship between the domesticated silkworms maintained for the long term and BmNPV in the surrounding mulberry fields, I sequenced the complete genome of BmNPV isolated from Hakozaki (referred to here as the Hakozaki strain).

The virus was amplified in NIAS-Bm-oyanagi2 cells by adding the hemolymph from a virus-infected larva to their culture medium (3), the budded virus (BV) was collected from the culture supernatant of infected cells by PEG precipitation, and viral genomic DNA was extracted from the collected BV by phenol-chloroform extraction and isopropyl alcohol precipitation.

A library was constructed from the extracted viral genomic DNA (250 ng) without performing shearing and size selection, using the Ligation Sequencing Kit (SQK-LSK109), following the manufacturer’s protocol, and the library was sequenced on an Oxford Nanopore Technologies MinION Mk1B using a Flongle Flow Cell (R9.4.1) for 24 h, producing 19.6 k reads with a minimum read length of 1,000 bp. MinKNOW 23.07.5 was used to collect the raw data and the Guppy 6.5.7 to perform basecalling with super-accuracy model. The Nanopore read *N*_50_ was 23,006. Following basecalling, DNA sequences were directly utilized and *de novo* assembled using the Flye 2.9.2 with the “–asm-coverage 50” option (4) and polished with the medaka 1.8.0. The assembly resulted in a single, circular contig, with a final genome read coverage of 72. Finally, the complete genome sequence was annotated by blastn with CDS sequences of BmNPV T3 strain [GenBank accession number L33180] as reference (5) and manually curated.

The genome of BmNPV Hakozaki strain consists of 127,783 bp with a G + C content of 40.3% and contained 142 open reading frames (ORFs), including ORFs for the 38 core genes of *Baculoviridae* (6, 7). In addition, the genome is 98.82% and 98.95% identical to those of BmNPV T3 and H4 [GenBank accession number LC150780] isolated from Japan (8) and has four *bro* genes (*bro-a*, *bro-b*, *bro-c*, and *bro-d*) and eight *hr* regions (*hr1*, *hr2L*, *hr2R*, *hr3*, *hr4a*, *hr4b*, *hr4c*, and *hr5*). However, BmNPV Hakozaki lacks the *bro-e* gene that are commonly found in BmNPV T3 and H4.

## Data Availability

The genome sequence was deposited with DDBJ/EMBL/GenBank under the accession number LC790006, and the raw read was deposited with the DDBJ Sequence Read Archive (DRA) under the BioProject number PRJDB17109.
